# The Impact of Dynamic Effects on the Results of Non-Destructive Falling Weight Deflectometer Testing

**DOI:** 10.3390/ma17174412

**Published:** 2024-09-07

**Authors:** Paweł Tutka, Roman Nagórski, Magdalena Złotowska

**Affiliations:** Faculty of Civil Engineering, Warsaw University of Technology, Al. Armii Ludowej 16, 00-637 Warsaw, Poland; roman.nagorski@pw.edu.pl (R.N.); magdalena.zlotowska@pw.edu.pl (M.Z.)

**Keywords:** dynamics, inertia force, pavement structure, falling weight deflectometer, backcalculation, numerical modelling

## Abstract

The article investigates the impact of applying a dynamic computational model that considers inertia forces on pavement deflections under rapidly changing loads over time. This study is particularly relevant to the modelling of falling weight deflectometer (FWD) testing. Initially, the article examines the deflection values obtained from computational models under loads with varying frequencies. In this context, considering inertia forces was significant for load durations shorter than 0.04 s. In such cases, the results of static and dynamic analyses differed considerably. One application of FWD measurement results is determining the stiffness moduli of pavement layers using backcalculation. The study explored the impact of incorporating inertia forces into the pavement model on the estimated values of stiffness moduli obtained via backcalculation. The results revealed differences of several percent between the stiffness moduli calculated using dynamic and static numerical models. Subsequently, the key pavement deformations and fatigue life were determined using the obtained moduli. Again, significantly different results were observed between dynamic and static cases. Based on these findings, it can be concluded that dynamic effects should not be ignored when using FWD testing for backcalculation. Additionally, the article addresses the sensitivity of backcalculation results, which is crucial for the accurate interpretation of the obtained data.

## 1. Introduction

Regular pavement-condition assessments are essential for effective road infrastructure management. These assessments enable the planning and execution of necessary repairs and reconstructions of damaged sections of the road network. One of the fundamental non-destructive diagnostic tests for determining pavement-bearing capacity is the falling weight deflectometer (FWD) test [1]. During this test, pavement deflections are measured under a known, stationary load applied over a short period. The measured values are the deflections of the pavement at selected measurement points, which are treated as indicators of pavement condition using the so-called deflection method. Additionally, the available data allow for the in situ determination of the stiffness moduli of the pavement structure layers. These calculated moduli are utilized, among other purposes, in the design of pavement reinforcements and the estimation of remaining life using mechanistic-empirical methods [2,3,4,5].

The method used to determine the stiffness of pavement layers is backcalculation. In an iterative process, a set of stiffness moduli is determined, for which the measured deflections are matched under the considered pavement loading conditions according to the adopted computational model [6,7,8,9]. The thickness of individual structural layers is typically assumed based on separate measurements taken from core samples. To obtain reliable backcalculation results, one of the key elements is adopting an appropriate mechanical model of the pavement and making correct computational assumptions. An important aspect is the inclusion of inertia forces in the motion equations. Due to the significantly easier implementation and shorter computation time, the deflection state under a given load is often calculated assuming that the load is applied sufficiently slowly. This implies that there is no need to consider inertia forces during these calculations. This assumption may be adequate for pavement loading by a heavy vehicle wheel for typical speed; however, it becomes questionable when analysing non-destructive testing under impact loading, such as in the FWD test. Due to inertial delay and the finite propagation time of the deformation wave, the pavement reaches its deflection from the load after a certain period. Furthermore, due to the rapid variability of the load, the force at the load surface significantly decreases before the deformation wave reaches a medium located at a certain distance. These effects can only be accurately captured in a dynamic model. Therefore, understanding the differences resulting from the application of these two approaches becomes essential. Numerous studies [10,11,12,13] have demonstrated significant differences between models that consider inertia forces (referred to as dynamic models) and those that do not (referred to as static models). Despite this, simplified models that neglect inertia forces in the pavement are still commonly used in many backcalculation programs [9], even in the presence of dynamic impact loads. In recent years, the number of solutions allowing for the consideration of dynamic effects in loaded pavements has steadily increased [14,15,16,17]. On the other hand, solutions are also being developed to modify the measured dynamic deflection basin to make the static model suitable for backcalculation, thereby improving the calculation results [18]. As evidenced by the interest of researchers, the issue of accounting for inertia forces in pavement modelling during backcalculation based on FWD data is significant However, there is a lack of comparisons between the results of backcalculations obtained from dynamic and static models, including sensitivity analysis, that takes into account not only the obtained stiffness modulus values but also key deformations and fatigue life, which are crucial for structural design. Additionally, it should be noted that the presence of inertia forces is not the only important issue related to the use of FWD results. For example, the method of considering displacement continuity between layers [19] and the temperature of the asphalt layers [20] also significantly impact the deflection calculation results based on the numerical model. These other issues will not be addressed in this article.

As mentioned earlier, another crucial element influencing both static and dynamic calculations is the sensitivity of backcalculation [21,22]. This is a key issue in assessing the reliability of backcalculation results. This phenomenon causes high variability in the resulting elastic modulus values for seemingly identical input deflection data. Moreover, this can have further consequences when using the calculated moduli in mechanistic-empirical methods. To effectively use FWD results in design, it is essential to understand the impact of deflection variability on the variability of key strains resulting from mechanistic analysis and, consequently, on the variability of fatigue life. These phenomena occur in both dynamic and static calculations. Therefore, it is necessary to identify them, quantify them, and determine any differences in their characteristics in both cases.

The aim of this article is to analyse the impact of incorporating inertia forces into the equations of motion for road pavement when calculating deformations under dynamic loading, with a particular focus on the FWD test. The objective is to highlight the differences in results obtained from static and dynamic models in forward calculations, backcalculations, and sensitivity analyses. Firstly, the study identifies the differences resulting from adopting a static instead of a dynamic computational model for forward calculation. Then this research, which contributes to its originality, estimates the impact of different computational models on backcalculation results, along with the estimation of fatigue life based on the obtained stiffness moduli. Moreover, this was conducted while considering the sensitivity of backcalculation results to small changes in input data. Not only was the sensitivity of the resulting stiffness moduli analysed. Additionally, as a novel element, the sensitivity analysis for key strains and estimated fatigue life was presented, divided into static and dynamic calculation cases. The influence of random deflection measurement errors at individual points on the obtained stiffness moduli was also examined using correlation coefficients and a machine-learning method in the form of a Random Forest model [23]. In creating the numerical model used for calculating displacement and strain states, the so-called infinite elements were employed [24]. These elements ensure the effect of a quiet boundary and mitigate the reflection of waves from the boundary of the finite element area. The occurrence of wave reflection significantly alters the obtained deflection results and may lead to incorrect comparisons between static and dynamic cases.

## 2. Materials and Methods

### 2.1. Pavement Subjected to Rapidly Changing Load

Firstly, the method for calculating pavement deflections under a given load and material properties will be discussed, as well as the method for examining the impact of dynamic effects on the obtained results. In the FWD study, the force applied to the pavement surface is of an impact nature, with a load duration ranging from 0.02 s to 0.06 s. The load surface is circular with a radius of 15 cm, the resultant load force is 50 kN, and the load position remains constant. The maximum deflections of the pavement over time are measured. Initially, the paper analyses the deflection values obtained from FWD test simulations, depending on the adopted numerical model and loading time. The study considered a load intensity described by a sinusoidal function (which is an idealization of the load applied in FWD tests) applied to a circular surface with a radius of 15 cm: *p*(*t*) = *p*_0_ sin (π *t*/*T*). Five load durations *T* were considered, corresponding to load frequencies *f* according to the formula *f* = 1/(2*T*). Values of parameters are presented in Table 1.

Calculations were performed for four pavement models in the form of a layered half-space (composed of homogeneous and isotropic layers). The models differed in the way inertial forces were considered and the method of modelling the material properties of asphalt layers. The applied models are presented in Table 2. The non-asphalt layers were assumed to be linearly elastic (Hooke’s model). It was assumed that there is full bonding between the layers of the road pavement.

**Figure 1:**
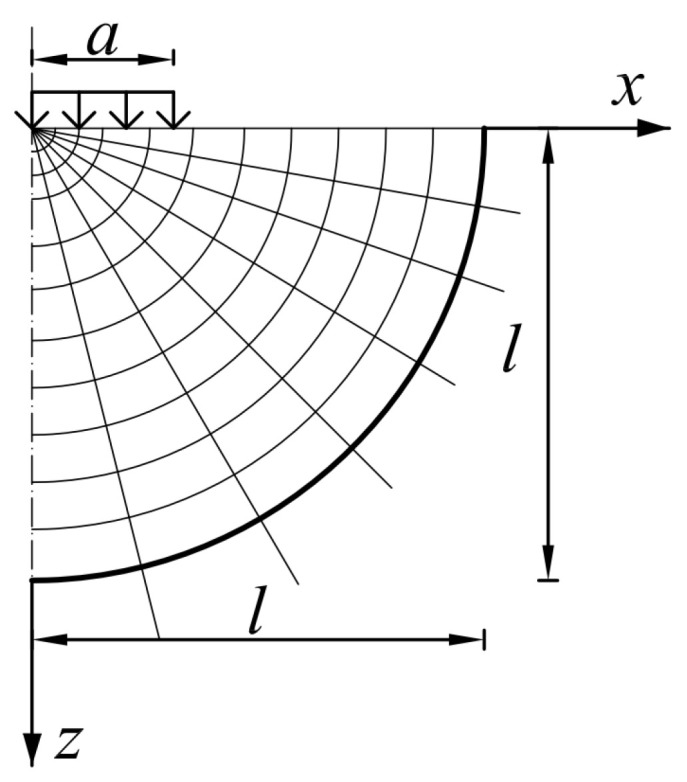
FEM domain of pavement computational modelling.

In order to perform calculations using the FEM method and Abaqus software (version 6.14) [24], an axisymmetric model in the shape of a hemisphere (due to the axisymmetric nature of the load) was adopted, using elements with linear shape functions CAX4R and CAX3, as well as infinite FEM elements CINAX4 [24] to prevent wave reflection from the edge of the modelled area (Figure 1). The radius of the area composed of CAX4R and CAX3 elements is 6 m, and additionally, infinite elements were placed on the perimeter of the finite element area [25].

The stiffness modulus measurement results for the mineral-asphalt mix were obtained from [26]. The stiffness of the mixes was measured according to the EN-12697-26 standard [27], Annex B (prismatic beams). Measurements were taken at temperatures of −2, 10, and 23 °C and at loading frequencies of 0.1, 0.2, 0.5, 1.0, 2.0, 5.0, 8.0, 10.0, 20.0, and 30.0 Hz. The obtained measurement results are presented in Table 3. All calculations will be carried out assuming a temperature of 10 °C.

**Figure 2:**
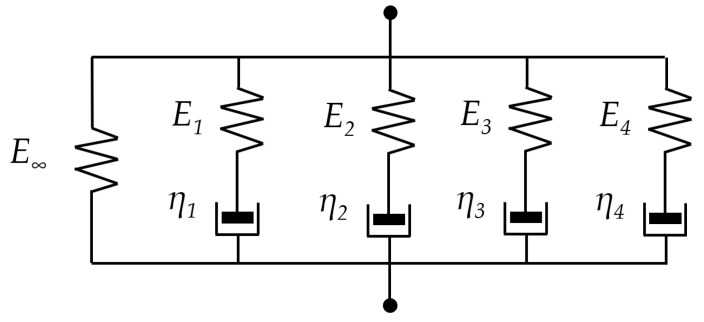
Diagram of a 5-branch Maxwell model.

These results were used to determine the parameters of a five-branch Maxwell model, which is implemented as a Prony series (Figure 2). The relationship between the complex modulus and loading frequency is expressed by Equation (1).


(1) $$E^{*}\left(\omega \right)=E_{\infty }+\sum_{i=1}^{n}\frac{E_{i}\omega^{2}\tau_{i}^{2}}{1+\omega^{2}\tau_{i}^{2}}+i\left(\sum_{i=1}^{n}\frac{E_{i}\omega \tau_{i}}{1+\omega^{2}\tau_{i}^{2}}\right)$$

In Abaqus, the Maxwell model is defined using the dimensionless parameters *g_i_* and *τ*. The relationship between these parameters and the quantities shown in Figure 2 is presented by Equations (2)–(4). By fitting the master curve to the data from Table 3, a set of parameters that best describes the experimentally observed results was found. The obtained parameter values are presented in Table 4.


(2) $$E_{0}\;=\;E_{\infty }\;+\;E_{1}\;+\;E_{2}\;+\;E_{3}\;+\;E_{4}$$

(3) $$g_{i}\;=\;\frac{E_{i}}{E_{0}}$$

(4) $$\tau_{i}\;=\;\frac{\eta_{i}}{E_{i}}$$

The obtained parameters were used in Abaqus for the viscoelastic model and to calculate the dynamic stiffness for a given load duration using the linear elastic material model (Hooke’s Law). A summary of all material properties used in the study, regardless of the type of model, is presented in Table 5 (*ρ*—mass density, *ν*—Poisson’s ratio, *E*—Young Moduli). For the MS2 and MD2 models, the stiffness of the asphalt layer was modelled as a viscoelastic material using the Maxwell model (parameters as shown in Table 4). For the MS1 and MD1 models, where the asphalt layers were modelled as linearly elastic, the elastic modulus was taken as the dynamic modulus corresponding to the load frequencies. Calculations were conducted on a pavement structure comprising three layers with distinct material properties: an asphalt layer, a base layer of aggregate, and a subgrade soil layer. The reason for such layer aggregation is the high sensitivity of the backcalculation results [21,22]. The variability in the elastic modulus values of the asphalt layers, which produced almost identical deflection basins, was so large that it was deemed more reliable to aggregate the asphalt layers with similar stiffness into a single layer and to model the base layer of aggregate with the improved subgrade. The following values of Poisson’s ratios and mass densities for the mentioned layers were assumed, as presented in Table 5.

### 2.2. Backcalculation Scheme

In the second part of the paper, the results of elastic modulus calculations for pavement layers using the backcalculation method were obtained. The calculations were performed using the MS1 and MD1 models. In both models, the layer materials were assumed to be linearly elastic (Hooke’s model), and the pavement was modelled as consisting of three layers. The result of backcalculation is the stiffness modulus, which is treated as dynamic moduli at a given loading frequency and as elastic moduli in Hooke’s models. Linear elastic models were used for backcalculation due to the sensitivity of calculations and the fact that the pavement was subjected to a force with a constant frequency during the test. This complicates obtaining reliable parameters for viscoelastic models based on FWD test data.

**Figure 3:**
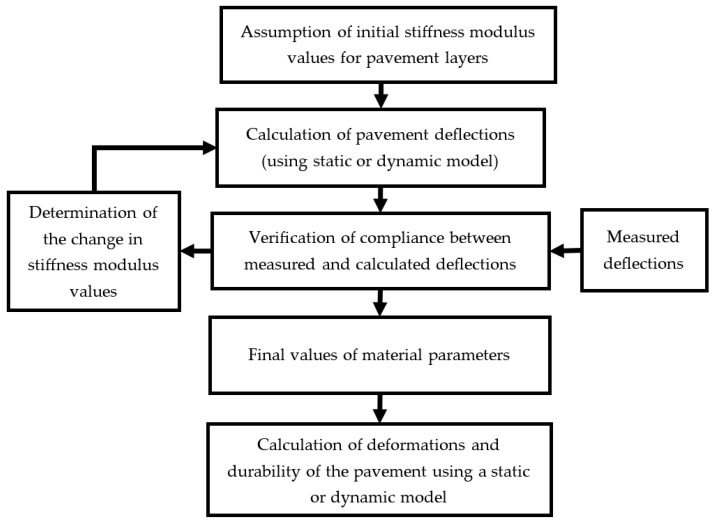
Backcalculation scheme.

The backcalculation involves computing pavement deflections for successive assumed values of elastic moduli until the computed deflections match those measured in the field test. The first step was to assume initial values for the elastic moduli and perform deflection calculations. The calculated deflections were compared with those obtained from measurements. If the difference exceeded the set threshold for material parameters, the values were adjusted according to the predefined procedure. Calculations were then redone with the new set of material parameter values. This process was repeated until the calculated deflections were consistent with the measured deflections, within the assumed level of agreement. The secant method was used to determine subsequent elastic modulus values. The root mean square (RMS) difference was used as the function defining the discrepancy between measured and calculated deflections (a termination condition for the calculations was that the root mean square error be less than or equal to 0.5 micrometres). The backcalculation process is illustrated in Figure 3 and is described in detail in reference [22]. Backcalculations based on pavement deflections during the FWD test were performed for both the MS1 and MD1 models to compare the results obtained and assess the impact of including inertial forces. Finally, the obtained moduli results were used to determine the values of key deformations and the fatigue life of the pavement.

In the backcalculation process, it is necessary to determine the pavement deflections for a given pavement model and its loading (MS1 and MD1 models with dynamic moduli for a loading time of 0.03 s). In this study, these calculations were performed for a specified deflection basin—referred to as the measured deflections—with values of 280 µm, 252 µm, 236 µm, 189 µm, 150 µm, 119 µm, and 96 µm at measurement points located at distances of 0 cm, 20 cm, 30 cm, 60 cm, 90 cm, 120 cm, and 150 cm from the centre of the loading surface (deflections denoted as *u*_1_ through *u*_7_, where *u*_1_ is the deflection at the load axis). This deflection basin corresponds to the actual deflections obtained during FWD measurements. The pavement calculations were carried out using FEM with Abaqus software (version 6.14) [24] for a load duration of *T* = 0.03 s, with a maximum wheel load force on the pavement of *P*_0_ = 50 kN uniformly distributed over a circular surface with a radius of *a* = 0.15 m and varying intensity over time according to the function *p*_0_ sin(π*t*/*T*), where *p*_0_ = 707 kPa. The loading time of 0.03 s is commonly used in FWD tests. It is often assumed to correspond to the typical speed of heavy vehicles (60 km/h).

After determining the elastic modulus of the asphalt layers and the remaining layers (unbound base and subgrade), the obtained results were used to calculate the key deformations necessary for determining pavement fatigue life. The maximum tensile strains at the bottom of the asphalt layers and the maximum compressive strains at the top surface of the subgrade were calculated. These values were determined under a wheel load of 50 kN, uniformly distributed over a circular area with a radius of *a* = 13.54 cm, at a tire pressure of 850 kPa. The pavement model used for these calculations is nearly identical to the one used for the elastic modulus calculations obtained through backcalculation, with the only differences being the values of the moduli and the method of load application. The calculations of these deformations, referred to as critical strains, were performed using the static model, which yields almost identical results to the dynamic model in the case of vehicle wheel loading traveling at typical speeds. Subsequently, the obtained strains were converted into fatigue life with respect to bottom-up fatigue cracking according to the criteria in [28] and structural rutting fatigue life based on the Asphalt Institute formulas [29].

## 3. Results

### 3.1. Computed Deflection Results for Rapidly Changing Loads

**Figure 4:**
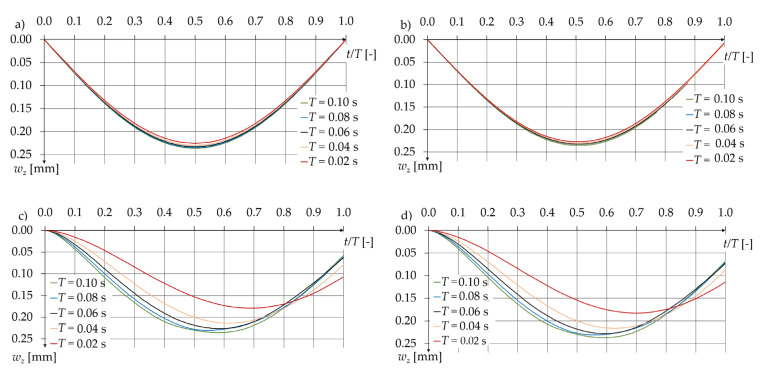
Deflection at the upper surface point: x = 0 and y = 0, as a function of time (**a**) model MS1 (**b**) model MS2 (**c**) model MD1 (**d**) model MD2.

Initially, the deflections of the pavement under rapidly changing (impact) loads were analysed for four computational models: MS1, MS2, MD1, and MD2. The analysis of the calculation results allowed for determining the influence of inertia forces in the pavement on the deflections. In the case of dynamic models, regardless of the material property considerations, pavement deflections decreased significantly as the loading time *T* decreased. Part of the decrease in deflections is attributed to the viscoelastic properties of the asphalt layers, while a larger portion of the decrease in deflection values at shorter loading times is due to the finite propagation time of the deformation wave through the pavement structure. This is evident when comparing MD1 with MS1 (Figure 4a,c) and MD2 with MS2 (Figure 4b,d), where deflections differ markedly despite identical material property modelling approaches. The use of a dynamic model captures the phenomenon that under very rapid load changes, the intensity starts to decrease before the wave propagates through the medium. This has a noticeable impact on the calculation results, which are summarized in Table 6 and depicted in Figure 4. Additionally, Figure 4c,d show the displacement of maximum deflection over time due to material inertia. The time-displacement effect in the MD1 and MD2 models for short loading times (*T*) is greater than the time displacement caused by the viscoelasticity of the asphalt layers observed in the MS2 model.

**Figure 5:**
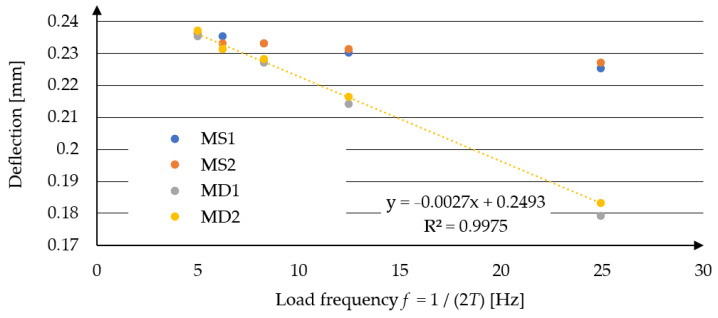
Maximum pavement deflection as a function of loading frequency—models MS1, MS2, MD1, MD2, and linear approximation for model MD2.

In Figure 5, the calculation results for all considered models are presented along with the formula defining the relationship between maximum deflection and loading frequency *f*, using model MD2. The results for MD1 and MD2, as well as MS1 and MS2, are nearly identical. Much larger discrepancies arise from including inertia forces in the motion equations. This significantly reduces the deflection values under short-duration loads.

### 3.2. The Impact of Dynamic Effects on Backcalculation

#### 3.2.1. Calculation Results—Elastic Modulus Values

The results of backcalculations using the dynamic model (MD1) and static model (MS1) were compared. Additionally, the variability in elastic modulus calculation results due to computational sensitivity was considered. This simulates variations in pavement deflection measurements, such as those caused by measurement errors. The determined elastic modulus values of pavement layers are highly sensitive to input data—pavement deflections. A relatively small change in deflections can lead to vastly different elastic modulus values. In this study, error was generated randomly using the Monte Carlo method with a normal distribution of mean zero and standard deviation *δ_i_* = (0.002 + 0.01*u_i_*)/3—where *u_i_* represents the deflection value at each *i*-th point in millimetres. Errors were independently generated for each deflection calculation, totalling one hundred cases.

**Figure 6:**
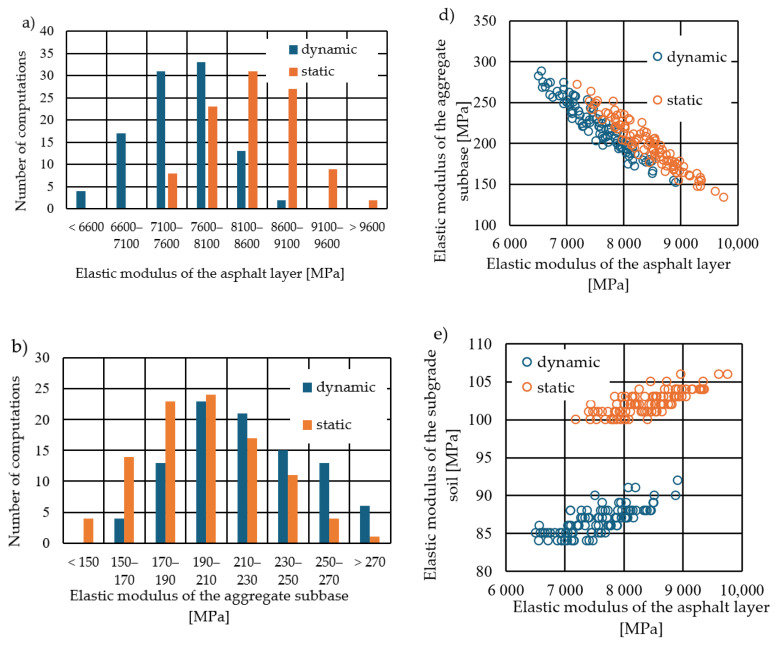

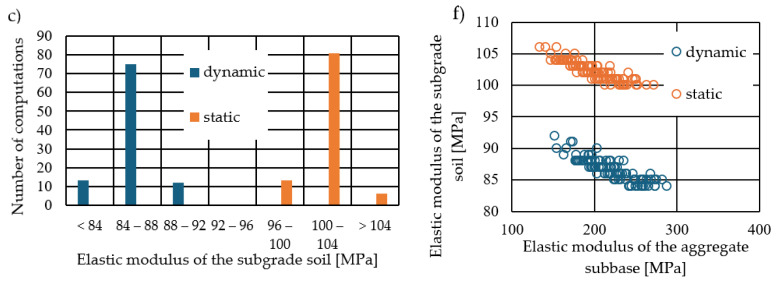
Histogram of obtained elastic moduli using static and dynamic calculations for (**a**) asphalt layers, (**b**) aggregate subbase, and (**c**) subgrade soil. Obtained elastic modulus values for (**d**) asphalt layers and aggregate subbase, (**e**) asphalt layers and subgrade soil, and (**f**) aggregate subbase and subgrade soil.

In Figure 6a, elastic modulus values of the asphalt layer obtained from calculations using the dynamic and static models are presented, assuming identical input deflections. Higher elastic modulus values were observed in the static calculations. This occurs because, in static calculations, the deflection values obtained (using the same material parameters) are greater than those in dynamic calculations (as seen in Figure 5). Therefore, to achieve the same deflection basin as in the dynamic case, higher elastic modulus values are required in the static case. Elastic modulus values were also determined for the aggregate subbase and subgrade layers. Figure 6b shows histograms of the obtained aggregate subbase elastic modulus values, and Figure 6c shows histograms of the calculated subgrade elastic modulus values for both dynamic and static calculations. Additionally, elastic modulus values for individual layers are presented in Figure 6d–f. When analysing the results, a change in the stiffness ratio between the respective moduli is observed. For dynamic calculations, the average ratio is *E*_b_:*E*_a_ = 2.9%, whereas for static calculations, it is 2.4%. This change in ratio is due to the slight difference in the shape of the deflection basins between static and dynamic calculations. The backcalculation results are sensitive to the shape of the deflection basins, as demonstrated later in the text, where the variability of the results is discussed.

#### 3.2.2. Critical Deformation Values and Fatigue Life Predictions

**Figure 7:**
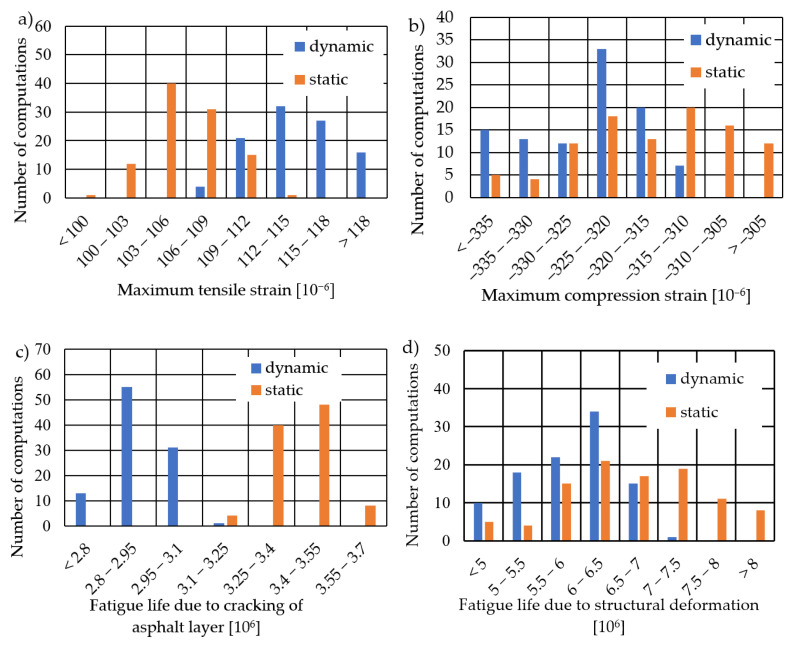
Histograms (**a**) of maximum tensile strains for dynamic and static calculations, (**b**) of maximum compressive strains for dynamic and static calculations, (**c**) of fatigue life due to fatigue cracking based on dynamic and static calculations, and (**d**) of fatigue life due to structural deformations based on dynamic and static calculations.

In this section, the strains under a load of 50 kN from a vehicle wheel were analysed for elastic moduli obtained from backcalculations. The calculations were performed using a model suitable for road pavement design, not for simulating FWD testing. Material properties were adopted as obtained from the backcalculations described in Section 3.2.1. Figure 7a shows the maximum tensile strain values obtained at the bottom of the asphalt layers. For the elastic modules from backcalculations using the dynamic model, the average value of maximum. tensile strain was found to be 114.7 × 10^−6^. For the elastic modules from backcalculation using the static model, this value was 106.2 × 10^−6^. The tensile strain values were used to calculate the fatigue life of the pavement due to fatigue cracking of the asphalt layer, based on empirical formulas from AASHTO 2004 [28], assuming the physical parameters of this layer as follows: asphalt volume content *V*_a_ = 9.86%, void volume content *V*_v_ = 7.00%, and a surface cracking index at 10%. Figure 7c presents histograms of pavement fatigue life for constructions using elastic modules based on static and dynamic calculations. When using the MS1 model in backcalculation, the predicted pavement fatigue life is 17.2% higher compared to using the MD1 model. This overestimation of remaining fatigue life is particularly disadvantageous. Figure 7b presents histograms of maximum compressive strain values on the surface of the subgrade. For the dynamic elastic modules, the average compressive strain was calculated as −325.1 × 10^−6^, whereas for the static elastic modules, the average value of maximum compressive strain was −316.8 × 10^−6^. The fatigue life was calculated for these strains due to structural fatigue deformations according to the formula from the Asphalt Institute [29]. Once again, using elastic modules calculated with dynamic models results in a lower estimated fatigue life.

In Table 7, the results of FWD simulation using backcalculation with dynamic and static models are summarized. The use of the static model inflates the elastic modulus values obtained from backcalculation. Using these inflated modulus values leads to an underestimation of the critical strain values, thereby overestimating the calculated fatigue life. The difference in fatigue life due to asphalt-layer cracking, which in this case determines the overall fatigue life, was 0.5 million 100 kN axle loads for the analysed structure. This results in a relative error of 17.2%. Such discrepancies can lead to significant errors in designing reinforcements based on FWD tests.

Additionally, to further analyse the sensitivity of the calculations, the variability of the input results was compared with the variability of the backcalculation outcomes. Coefficients of variation, defined as the ratio of the standard deviation to the mean value expressed as a percentage, were calculated. The variability in deflections, ranging from 0.6% to 1.0%, leads to significantly greater variability, especially in the resulting values of the asphalt layer elastic modulus (*E*_a_) and the subbase layer elastic modulus (*E*_b_). It is evident that even a small change in deflections (resulting from errors considered acceptable during FWD testing) leads to a significant variation in the elastic modulus results. The obtained values are presented in Table 8.

In the sensitivity analysis of the results, correlation coefficients (*ρ*) were calculated between the elastic modulus outcomes and the input deflections (Table 9). These coefficients indicate the influence of randomly selected deflection values on the elastic moduli obtained through backcalculation. Additionally, a model was developed using the Random Forest method [23], a machine-learning technique commonly used for regression. The model is constructed by training multiple decision trees on various subsets of data [23]. The metric indicating the influence of individual input variables on the outcomes is known as feature importance. These values represent the relative impact of each input variable on the model’s predictions, with higher values indicating a greater influence of a particular variable on the model’s outcome. In this context, a higher coefficient signifies a stronger influence of a specific deflection on the elastic modulus. The results derived from both dynamic and static models are presented in Table 10.

The elastic modulus of the asphalt layer is most strongly influenced by the deflection measured at the first point (*u*_1_). Correlation analysis reveals that an increase in *u*_1_ is associated with a decrease in the resulting elastic modulus of the asphalt layers. Positive correlations were found between the elastic modulus *E*_a_ and the deflections measured at the 3rd and 4th points. Conversely, an inverse relationship was observed in the correlation coefficients between the base course elastic modulus and deflections. In the performed calculations, higher deflections at *u*_1_ resulted in a higher base course modulus (*E*_b_), while higher deflections at *u*_3_ and *u*_4_ led to a lower *E*_b_ modulus. This indicates that the shape of the deflection basin significantly influences the elastic modulus results. Specifically, a steeper deflection basin resulted in higher values of the aggregate base course elastic modulus and lower values of the asphalt layer elastic modulus. In contrast, a flatter deflection basin led to lower aggregate base course modulus values and higher asphalt layer modulus values. Sensitivity to the shape of the deflection basin is the primary reason for the strong relationship observed between *E*_a_ and *E*_b_. Even small changes in the basin shape, which was considered in the study, cause significant variations in *E*_a_ and *E*_b_ values. The correlation coefficient between these two parameters for dynamic calculations is −0.96.

In the case of the subgrade soil elastic modulus (*E*_c_), it is more influenced by the deflections measured at the points located farther away. For both static and dynamic calculations, the obtained *E*_c_ modulus is most dependent on the deflection at *u*_7_ (Table 10). An increase in *u*_7_ is associated with lower *E*_c_ values (Table 9). When comparing the static and dynamic results, the outcomes are qualitatively very similar, with some differences arising from the slight variation in the shapes of the deflection basins.

## 4. Discussion

The article analysed the impact of incorporating inertia forces into the equations of motion on backcalculation results from falling weight deflectometer (FWD) testing and their subsequent influence on critical strain outcomes for flexible pavement design. These outcomes, calculated from elastic moduli obtained through backcalculation, directly affect predictions of pavement fatigue life. The study also examined maximum deflection values for various loading times across four pavement computational models, each differing in their representation of asphalt layer material properties and the consideration of inertia forces. Both of these factors potentially influence the computed values and the relationship between load frequency and resulting deflection outcomes. This analysis is particularly crucial for non-destructive diagnostic tests such as FWD testing, as using an incorrect model in backcalculation can lead to erroneous results in determining the elastic moduli of pavement layers. The calculations confirmed several conclusions previously reported in the literature:

- When dealing with rapidly varying loads, it is crucial to use a dynamic model that incorporates inertia forces due to the finite propagation time of deformation waves in the pavement. This is particularly significant for loading durations of 0.04 s or less.
- The use of a linearly elastic model for asphalt layers, employing the dynamic modulus as the elastic modulus, can lead to differences in calculation results compared to a viscoelastic model (though not as significant as when dynamic effects are omitted altogether). This discrepancy arises from the challenge of accurately determining the material’s load frequency when assessing the dynamic modulus.
- Backcalculation for FWD tests should be performed using a dynamic model. The use of a static model introduces significant discrepancies in the results.
- In diagnostic tests, results obtained within the loading duration range of 0.02 to 0.06 s should be normalized due to the duration of force application. Deflection calculation results for the same pavement and maximum load can vary significantly depending on the loading time; for example, the maximum deflection difference for the MD2 model is 25% between calculations for loading durations of 0.02 s and 0.06 s. This is important when using the so-called representative deflection obtained during FWD tests as a measure of bearing capacity in road condition diagnostics.
- When applying backcalculation, the sensitivity of results to the measured deflection values is crucial. Similar input data can lead to different combinations of material parameters, subsequently affecting critical strains and pavement fatigue life.

Furthermore, the results presented in the article justify the formulation of additional conclusions:

- Analysing the sensitivity of fatigue life results due to bottom-up asphalt layer cracking and permanent deformation shows significantly greater sensitivity in the latter case (with a coefficient of variation of 13.7% for static and 10.5% for dynamic calculations). This is due to the amplification of strain variability through the fatigue life equation, which involves approximately the fourth power of the strain. The variability in the fatigue life of asphalt layers is somewhat mitigated by considering the stiffness of asphalt layers in the fatigue life formula.
- The elastic moduli of the asphalt layers and the base course exhibit particularly high sensitivity in the results. In the study, where errors were relatively small and generated randomly, these values are strongly negatively correlated (−0.97). This means that when one value is relatively high, the other is lower. A closer analysis of the correlation between individual deflections and moduli reveals that higher stiffness of the upper layers is obtained for flatter deflection basins, while higher stiffness of the base course occurs in steeper deflection basins. This suggests that the calculations are particularly sensitive to changes in the shape of the deflection basin. The variability in elastic moduli *E*_a_ and *E*_b_ was primarily influenced by deflections at the first four points (located at distances of 0 cm, 20 cm, 30 cm, and 60 cm from the load axis). This effect is similar in backcalculations performed using dynamic and static calculations.
- The subgrade soil’s elastic modulus depends primarily on deflections farther from the load axis. The importance coefficient from the Random Forest model is 30% for dynamic calculations and 43% for static calculations. It is important to note that the variability of the determined soil modulus is significantly lower than that of other layers, making it less sensitive to random measurement errors. However, there is a consistent difference between the soil’s elastic modulus obtained in backcalculation using static and dynamic models due to the different shapes of the deflection basin in the various computational models.
- Comparing the sensitivity between static and dynamic results does not reveal significant qualitative differences. In static model calculations, the shape of the deflection basin is slightly different from that in dynamic calculations, which slightly alters the numerical relationships between deflections and the outcome values in the backcalculation process.

The results provide a basis for proposing certain changes in the approach to conducting and processing FWD data. These guidelines could be used when exceptionally accurate results are required:

- From the perspective of backcalculation sensitivity within a single measurement point, multiple measurements should be conducted to determine the actual variability of deflection results, which could be used in further data processing. The number of measurements should depend on the observed variability and the required accuracy. The average value obtained from multiple measurements would be a more reliable figure for further calculations.
- When performing backcalculations, the observed variability in deflections from multiple measurements should be considered, and this variability should be modelled accordingly, similar to the approach used in this study.
- When performing backcalculations with determined errors, the result should include confidence intervals or envelopes for the elastic moduli. The outcome of backcalculation should provide ranges of elastic modulus values along with the dependencies between the elastic moduli of individual layers.

Additionally, it must be stated again that, from a physical perspective, considering dynamic phenomena is the natural approach. Inertia forces are elements of established physical theories. In certain cases where the load is applied sufficiently slowly, it may be possible to omit them. However, this should be considered an exception rather than a rule.

Furthermore, several research topics and potential improvements specifically related to the FWD testing and backcalculation methods discussed in this article have been identified and warrant further investigation:

- The calculations were performed for a pavement structure with given layer thicknesses. It would be advisable to generalize the conclusions to a broader range of possible cases.
- The numerical models used in the calculations require experimental validation. Moreover, the adopted method for obtaining stiffness moduli in backcalculations should always be verified by independent measurement methods.
- The study presents considerations on the impact of using static and dynamic models in backcalculation on the resulting elasticity moduli and, consequently, on how changes in these moduli affect the estimated remaining fatigue life of the pavement structure. A separate issue is the consideration of inertia forces in both the pavement structure and the heavy vehicle moving on the pavement during calculations to determine pavement deformations, which are then used in fatigue life calculations. Including this aspect would provide a more complete picture of the difference in results when using static versus dynamic models.
- The study of pavement behaviour with a high-stiffness material layer (“bedrock”) located at a relatively shallow depth beneath the road surface. In such pavements, the influence of dynamic effects may be qualitatively different due to the reflection of waves within the flexible medium from the stiff layer. In this scenario, deflections calculated using a dynamic model might be larger than those predicted by a static model.
- The use of time-dependent deflection values in backcalculation to determine the viscoelastic parameters of asphalt layer models. In theory, analysing the time history of deflection curves should allow for the determination of viscoelastic parameters. However, the challenge lies in the potentially heightened sensitivity of such calculations to variations in the measured deflections.
- Determination of other pavement structural parameters, such as layer thickness and Poisson’s ratios, where the sensitivity of the results to relatively small changes in input data remains a significant issue.
- The application of artificial neural networks for objective function minimization in backcalculation procedures.

## Figures and Tables

**Table 1:** Values of parameters defining the load method.

No	Parameter Description	Symbol	Value	Units
1.	The value of pressure at time t	*p*(*t*)	ranging from 0 to 707	kPa
2.	Maximum load pressure	*p*_0_	707	kPa
3.	Time loadings	*T*	five cases were considered: 0.1, 0.08, 0.06, 0.04, 0.02	s
4.	Time variable	*t*	variables ranging from 0 to T	s
5.	The radius of the circular load surface	*a*	0.15	m
6.	Load frequencies	*f*	five cases were considered: 5, 6.25, 8.33, 12.5, 25	Hz

**Table 2:** Considered pavement structure models.

No	Model Description	Model Designation	Inclusion of Inertia Forces	Modelling Approach for Asphalt Layer Properties
1.	Static Model	MS1	No	Linearly elastic model. Dynamic modulus at a given frequency of loading as the elastic modulus of the layer.
2.	Quasi-Static Model	MS2	No	Viscoelastic model. Properties are defined using a Prony series.
3.	Dynamic Model	MD1	Yes	Linearly elastic model. Dynamic modulus at a given frequency of loading as the elastic modulus of the layer.
4.	Dynamic Model	MD2	Yes	Viscoelastic model. Properties are defined using a Prony series.

**Table 3:** Elastic stiffness of asphalt layers depending on the frequency of loading (at 2 °C, 10 °C, 23 °C).

No.	Frequency [Hz]	*E* [MPa] for −2 °C	*E* [Mpa] for 10 °C	*E* [Mpa] for 23 °C
1.	0.1	13,748	7721	3357
2.	0.2	14,789	8546	3836
3.	0.5	16,044	9695	4576
4.	1.0	17,022	10,596	5206
5.	2.0	18,002	11,534	5900
6.	5.0	19,293	12,806	6904
7.	8.0	19,988	13,483	7465
8.	10.0	20,326	13,818	7728
9.	20.0	21,229	14,795	8607
10.	30.0	21,807	15,358	9124

**Table 4:** Parameters of the Maxwell model.

No.	Parameter Name	Parameter Value
1.	*E*_0_ [Mpa]	15,839.4
2.	*g*_1_ [1]	0.198833
3.	*g*_2_ [1]	0.177083
4.	*g*_3_ [1]	0.167500
5.	*g*_4_ [1]	0.455187
6.	*τ*_1_ [s]	0.011212
7.	*τ*_2_ [s]	0.082304
8.	*τ*_3_ [s]	0.664383
9.	*τ*_4_ [s]	97.0579
10.	RSME of *E*_d_ [Mpa]	41.09

**Table 5:** Geometric and material parameters of the adopted road pavement structure.

No	Layer	*h* [cm]	*E* [Mpa]	*ρ* [kg/m^3^]	*ν* [−]
1.	Asphalt layer	22	Dynamic modulus or Prony series—parameters are presented in Table 4.	2615	0.3
2.	Subbase made of unbound aggregate	20	300	2250	0.3
3.	Subgrade soil	∞	100	1800	0.35

**Table 6:** Maximum pavement deflection values based on four computational models for various loading times.

No.	Loading Time [s]	Deflection According to Model MS1 [mm]	Deflection According to Model MS2 [mm]	Deflection According to Model MD1 [mm]	Deflection According to Model MD2 [mm]
1.	0.10	0.236	0.236	0.235	0.237
2.	0.08	0.235	0.233	0.231	0.231
3.	0.06	0.233	0.233	0.227	0.228
4.	0.04	0.230	0.231	0.214	0.216
5.	0.02	0.225	0.227	0.179	0.183

**Table 7:** Comparison of elastic modulus results of pavement layers using dynamic and static analysis models in backcalculation.

No.	Parameter	Average Values—Dynamic Model	Average Values—Static Model	Relative and Absolute Difference Based on Dynamic and Static Analysis	
				Absolute Difference	Relative Difference [%]
Elastic modulus [MPa]	*E*_a_	7565	8423	858	11.3
	*E*_b_	221	198	−23	−10.1
	*E*_c_	87	102	15	17.2
Strains [10^−6^]	*ε*_h_	114.6	106.2	−8.4	−7.3
	*ε*_v_	−325.1	−316.8	8.3	−2.6
Fatigue life 100 kN axis [10^6^]	*N*_f_	2.91	3.41	0.50	17.2
	*N*_d_	5.89	6.65	0.76	12.9

**Table 8:** The obtained values of the coefficient of variation for the input and output data.

**No.**	**Parameter**	**Static Model [%]**	**Dynamic Model [%]**
Displacement [mm]	*u*_1_	0.6	0.6
	*u*_2_	0.7	0.7
	*u*_3_	0.6	0.6
	*u*_4_	0.7	0.7
	*u*_5_	0.7	0.7
	*u*_6_	0.9	0.9
	*u*_7_	1.0	1.0
Elastic modulus [MPa]	*E*_a_	6.5	7.2
	*E*_b_	15.0	14.1
	*E*_c_	1.5	2.1
Strains [10^−6^]	*ε*_h_	2.6	2.9
	*ε*_v_	−3.1	−2.4
Fatigue life 100 kN axis [10^6^]	*N*_f_	2.8	2.7
	*N*_d_	13.7	10.5

**Table 9:** Correlation coefficients between deflection values and the resulting elastic moduli.

	*E*_a_ Dynamic Model	*E*_b_ Dynamic Model	*E*_c_ Dynamic Model	*E*_a_ Static Model	*E*_b_ Static Model	*E*_c_ Static Model
*u*_1_	−0.78	0.63	−0.42	−0.76	0.59	−0.44
*u*_2_	0.08	−0.19	0.14	−0.03	−0.16	0.14
*u*_3_	0.36	−0.41	0.30	0.41	−0.56	0.44
*u*_4_	0.36	−0.36	0.14	0.73	−0.73	0.47
*u*_5_	0.18	−0.10	−0.13	0.44	−0.32	−0.05
*u*_6_	−0.02	0.14	−0.46	−0.06	0.24	−0.56
*u*_7_	−0.17	0.33	−0.53	−0.25	0.44	−0.72

**Table 10:** Importance values of variables for elastic moduli obtained from the Random Forest model.

	*E*_a_ Dynamic Model	*E*_b_ Dynamic Model	*E*_c_ Dynamic Model	*E*_a_ Static Model	*E*_b_ Static Model	*E*_c_ Static Model
*u*_1_	0.60	0.39	0.22	0.62	0.26	0.11
*u*_2_	0.05	0.10	0.06	0.01	0.02	0.02
*u*_3_	0.15	0.20	0.10	0.07	0.09	0.09
*u*_4_	0.11	0.12	0.05	0.25	0.41	0.25
*u*_5_	0.02	0.03	0.04	0.01	0.01	0.02
*u*_6_	0.03	0.07	0.23	0.01	0.03	0.08
*u*_7_	0.04	0.10	0.30	0.03	0.18	0.43
*∑*	1.00	1.00	1.00	1.00	1.00	1.00

## Data Availability

The original contributions presented in the study are included in the article, further inquiries can be directed to the corresponding author.

## Abbreviations

*p*(*t*)	the value of the pressure loading the pavement at time *t* [kPa]
*p*_0_	maximum load pressure [kPa]
*T*	loading time [s]
*a*	radius of the circular load area [m]
*f*	load frequency [Hz]
*ω*	loading angular frequency [rad/s]
*E**	complex modulus
*i*	imaginary unit
*E*_0_, *E*_∞_, *E*_1_, *E*_2_, *E*_3_, *E*_4_	parameters (stiffnesses) in the generalized Maxwell model [MPa]
*η*_1_, *η*_2_, *η*_3_, *η*_4_	parameters (viscous damper) in generalized Maxwell model [MPa·s]
*g*_1_, *g*_2_, *g*_3_, *g*_4_	dimensionless parameters in the generalized Maxwell model [-]
*τ*_1_, *τ*_2_, *τ*_3_, *τ*_4_	retardation times, parameters in generalized Maxwell model [s]
*E*_a_	elastic modulus of asphalt layer [MPa]
*E*_b_	elastic modulus of subbase [MPa]
*E*_c_	elastic modulus of subgrade [MPa]
*u*_1_, *u*_2_, *u*_3_, *u*_4_, *u*_5_, *u*_6_, *u*_7_	Deflections measured at the first, second, …, and seventh points during the FWD testing [mm]
*RSME*	root square mean error
*ρ*	density of material [kg/m^3^]
*ν*	Poisson ratio [-]
*ε*_h_	maximum tensile strain at the bottom of asphalt layer [10^−6^]
*ε*_v_	maximum compression strain at the surface of the subgrade [10^−6^]
*V*_a_	asphalt volume content of asphalt layer [%]
*V*_v_	void volume content of asphalt layer [%]
*N*_f_	the fatigue life of the pavement due to fatigue bottom-up cracking of the asphalt layer [mln of 100 kN axes]
*N*_d_	fatigue life due to structural fatigue deformations [mln of 100 kN axes]
